# Effect of the Different High Volume Fraction of SiC Particles on the Junction of Bismuthate Glass-SiC_p_/Al Composite

**DOI:** 10.1155/2018/7394040

**Published:** 2018-02-20

**Authors:** Bin Wang, Shengguan Qu, Xiaoqiang Li

**Affiliations:** ^1^Guangdong Key Laboratory for Processing and Forming of Advanced Metallic Materials, South China University of Technology, Guangzhou 510640, China; ^2^National Engineering Research Center of Near-Net-Shape Forming for Metallic Materials, South China University of Technology, Guangzhou 510640, China

## Abstract

The in-house developed bismuthate glass and the SiC_p_/Al composites with different volume fractions of SiC particles (namely, 60 vol.%, 65 vol.%, 70 vol.%, and 75 vol.%) were jointed by vacuum hot-pressing process. The novel material can be used for the space mirror. The SiCp is an abbreviation for SiC particle. Firstly, the SiC_p_/Al composites with different vol.% of SiC particle were manufactured by using infiltration process. In order to obtain a stable bonding interface, the preoxide layers were fabricated on the surfaces of these composites for reacting with the bismuthate glass. The coefficient of thermal expansion (CTE) was carried out for characterizing the difference between the composites and bismuthate glass. The sealing quality of the composites and the bismuthate glass was quantified by using shear strength testing. The optical microstructures showed the particles were uniformly distributed in the Al matrix. The SEM image shows that a smooth oxidation layer was generated on the SiC_p_/Al composite. The CTE testing result indicated that the higher the vol.% of the particles in the composite, the lower the CTE value. The shear strength testing result disclosed that SiC_p_/Al composite with relatively low CTE value was favorable to obtain a bonding interface with high strength.

## 1. Introduction

With the rapid development of aerospace optical systems, more and more aerospace researchers are paying their attention to the materials applied in large-scale space-based mirror [1]. As an important component for use in aerospace, the space-based mirrors have to satisfy the strict functional requirements with lightweight, sound dimensional stability, and good resistance to thermal shock. In addition, the usability of this mirror in the space environment mainly depends on the bond strength of the sealing quality between the glass and the substrate of the mirror. During the past decades, the most commonly used materials for space-based mirrors are beryllium [2], Zerodur [3], and ULE [4]. These glasses are usually installed to the metal frame (e.g., Invar or Kovar alloys) of the space mirror by using the special glues [5].

In order to decrease the cost and weight and increase the strength of this mirror and sealing reliability of the glass on the substrate of the mirror, SiC_p_/Al composites have been deemed as the candidate [6, 7]. As one of the most popular aluminum matrix composites (AMCs), SiC_p_/Al with high SiC volume fraction possesses high polishability, high specific strength, low density, and low coefficient of thermal expansion (CTE). After preparing the oxidation film (which can be obtained by using natural oxidation, anodizing or plasma electrolytic oxidation processes) on SiC_p_/Al composite, the sealing reliability of this mirror fabricated by the composites and glasses will be enhanced. In our previous study [8], a novel space mirror was developed by using the bismuthate glass sealed with the preoxidized SiC_p_/Al composite (70 vol.%  SiC). The shear strength of this composite-glass material reached 5.44 MPa which was about 20% higher than that of the popular borosilicate glass-to-Kovar alloy joint material [9].

The chemical reaction and elements diffusion at the interface between bismuthate glass and preoxidized SiC_p_/Al is favorable to the interfacial properties of glass-composite materials. Besides, the CTE value of SiC_p_/Al composite also plays an important role in the bonding of glass-composite. Both the CTE value and the characteristic of the oxidation layer on SiC_p_/Al composites are related to SiC volume fraction.

However, to the best of our knowledge, few works have been carried out on the association between the microstructure of SiC_p_/Al composites and the bonding property of the bismuthate glass and SiC_p_/Al composite material. In this study, the SiC_p_/Al composites with different SiC volume fractions were prepared and preoxidized to joint with bismuthate glass. The CTE and mechanical properties of these composites were also tested for the assessment of their application prospect.

## 2. Experiment

### 2.1. Fabrication of SiC_p_/Al Composites and Preoxidation of the Composites

**Figure 1:**
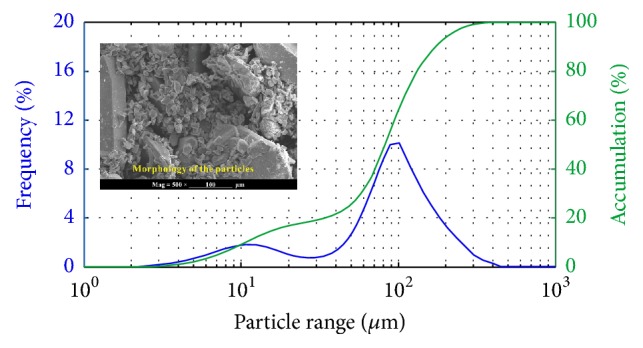
Morphologies and size distribution of SiC particles.

Table 1 shows the composition of the commercial 6061 aluminum alloy used as the matrix of this composite. The green silicon carbide powders with three different average particle sizes, 2 *μ*m, 8 *μ*m, and 128 *μ*m, were blended by ball milling process and used in present study.  Figure 1 shows the distributions of these SiC particles. It indicates the median diameters of these particles are about 2 *μ*m, 8 *μ*m, and 128 *μ*m. The colloidal silica (produced by Shanghai, Yanchen Industrial Co., Ltd, China) was carried out for the binder of SiC preform. The porous SiC particles preformed with the dimension of Φ 75 mm  ×  *H* 40 mm were sintered at 1600°C for 2 h.

The SiC_p_/Al composites with relatively high SiC volume fraction were fabricated via infiltration process. To begin with, the commercial 6061 alloy was melted in a graphite crucible at 750°C. When the temperature of molten aluminum alloy was declined to about 700°C (tested by the thermocouple), it was removed to a steel container (preheated to 500) in the hydraulic pressure machine (Huzhou machine tool plant, Co., Ltd, China). Thereafter, the Al melt was squeezed by the hydraulic machine and infiltrated into the SiC preform. The squeezing pressure was evenly changed from 8 MPa to 90 MPa during the solidification process of the composite (lasted for about 20 min).

**Figure 2:**
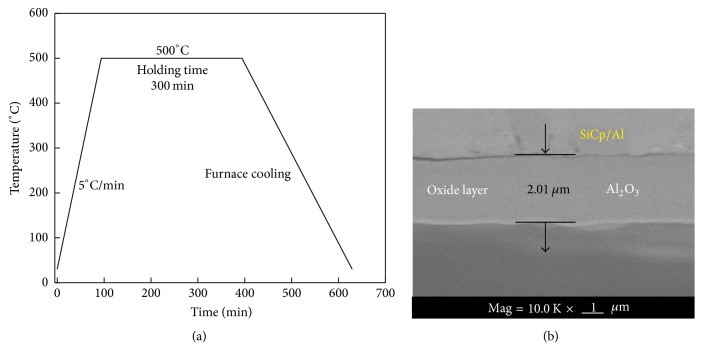
Preparation oxidation film of high volume fraction. (a) The process flow of preparing the oxidized layer on the composite and (b) the oxidized Al_2_O_3_ layer on the surface of the composites.

In our work, the SiC_p_/Al composite oxidation film was prepared in the air atmosphere at 500°C with the holding time of 300 min (see details in Figure 2(a)). The oxidized surface of the SiC_p_/Al composites was observed by SEM and given in Figure 2(b). It is clear that the thickness of this oxidized layer is about 2.01 *μ*m. The upper part of the Al_2_O_3_ layer is SiCp/Al composite.

### 2.2. Sealing of Bismuthate Glass and SiC_p_/Al Composites

The in-house developed bismuthate glass for the application of aerospace was prepared by using the reagent grade Bi_2_O_3_ (72.7 wt.%), B_2_O_3_ (15.0 wt.%), BaO (10 wt.%), and Li_2_O (2.3 wt.%) as shown in Table 2 [8]. The bismuthate glass was melted in a graphite crucible. Thereafter, this bismuthate glass was vacuum hot press combined with the fabricated composites with four different SiC volume fractions (60%, 65%, 70%, and 75%, respectively). In order to precisely combine the glass and the composites, both of them were grounded and polished, respectively, before conducting the vacuum hot press composite process. The Germany BMT (Breitmeier Messtechnik GmbH) measurement was carried out for measuring the roughness of the polished surface of the composites and glass. The SiC_p_/Al composites were polished to the roughness of Ra ≈ 0.15 *μ*m in a JP06A single-axle grinding polisher (using a 3000 mesh diamond wheel), whilst the bismuthate glass was polished to the roughness of Ra ≈ 0.025 *μ*m (using 500 mesh cerium oxide abrasive). Prior to oxidation, aluminum oxide on the surface of SiCp/Al was completely removed by rubbing, polishing, and ultrasonic cleaning and then blow-dried.

**Figure 3:**
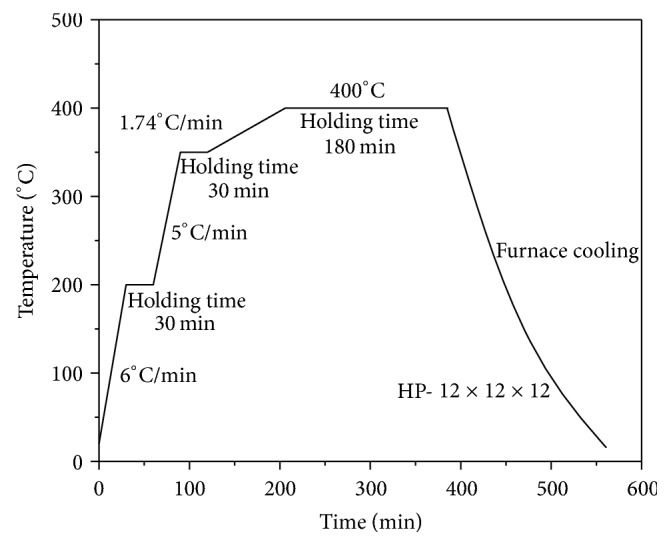
Process flow of the vacuum hot-pressing diffusion welding.

The diffusion welding for combining the glass and the composites was conducted by using the vacuum hot-pressing furnace (HP-12 × 12 × 12). According to the process flow of diffusion welding process given in Figure 3, the vacuum degree was set at ~5 × 10^−4^ Pa and the diffusion temperature was set at 400°C. The holding time of the diffusion welding process was set at 180 min. The reason is that the material is heated evenly and the elements can fully diffuse. When the diffusion welding was completed, the cross section of the combined material was machined out for the microstructure observation.

### 2.3. Coefficient of Thermal Expansion of the Glass and Composites

The sealing quality of bismuthate glass and the fabricated composites might be deteriorated by the strain incompatibility at their interfaces. Influenced by the difference of coefficient thermal expansion (CTE) at their interfaces, the strain incompatibility is likely to be produced during the cooling course after completing the sealing process. Hence, the CTE values of the glass and the fabricated composites with different volume fraction are necessary to know. The NETZSCH DIL402PC (with the working temperature from 0~1400°C) was carried out for the CTE testing. The glass and the fabricated composites were all machined out to cylindrical samples with the dimension of Φ 6 mm × 25 mm. Since the diffusion welding process was conducted at 400°C, the testing range of CTE value was set at the range of 25~400°C.

### 2.4. Shear Strength Measurement of the Sealing Interface

In order to know the bonding property at the glass/composite interface, shear strength testing (based on the standard of GB/T 12830-91) was conducted by using the Instron 3356 universal testing machine. The cylindrical glass with the dimension of Φ 75 mm × 15 mm was fabricated, and it was sealed by diffusion welding process on the SiC_p_/Al matrix. The nominal shear strength *τ* on the testing samples can be determined via

(1) $$\begin{array}{c} \tau =\frac{F_{max}}{S}, \end{array}$$

where *F*_max_ and *S* are the maximum shear and the effective bearing area of the sealed cylindrical sample, respectively. The effective bearing area *S* is about 4.41 × 10^-3 ^m^2^.

## 3. Results

### 3.1. XRD Analysis and Identification and Microstructure Observation

**Figure 4:**
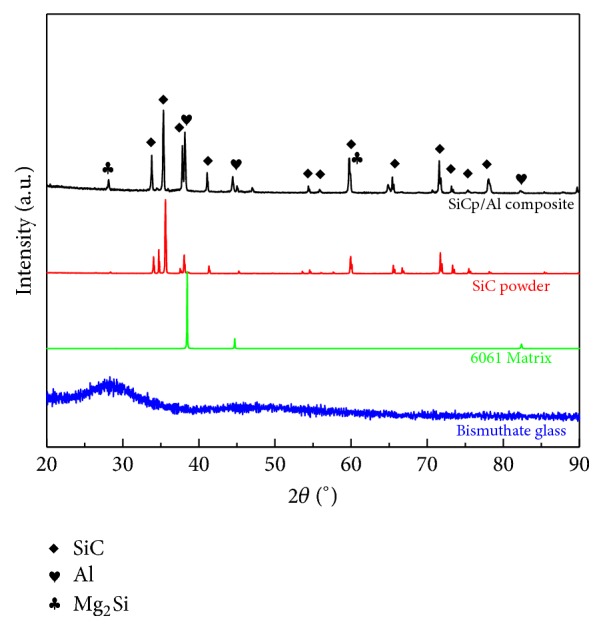
XRD patterns of the bismuthate glass, SiC powders, 6061Al matrix, and the fabricated SiC_p_/6061Al composites.

Figure 4 shows the XRD patterns of the bismuthate glass, the composite matrix, and the fabricated SiC_p_/6061Al composite. Obviously, no sharp peak is revealed in the pattern of bismuthate glass. It indicates that the glass did not undergo crystallization during the melting process and the powdery sample was amorphous. Compared with the Al matrix, the amount of the elements such Mg, Si, and Fe is very low. Hence, the intensity of the *α*-Al peaks is far more stronger than the others in the pattern of the composite matrix. All the peaks revealed in the pattern of the SiC powders are matched with the standard diffraction card. It also can be found that the peaks corresponding to Mg_2_Si phase are revealed in the SiC_p_/6061Al composite.

### 3.2. Microstructure of the SiC_p_/6061Al Composites

**Figure 5:**
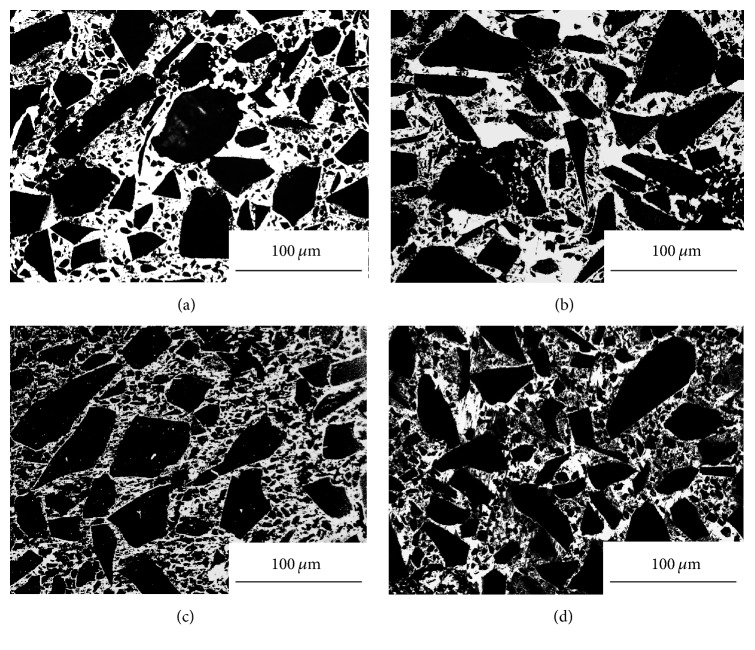
Optical microstructures of SiC_p_/Al composites with different additions of SiC particles: (a) 60 vol.%, (b) 65 vol.%, (c) 70 vol.%, and (d) 75 vol.%.

**Figure 6:**
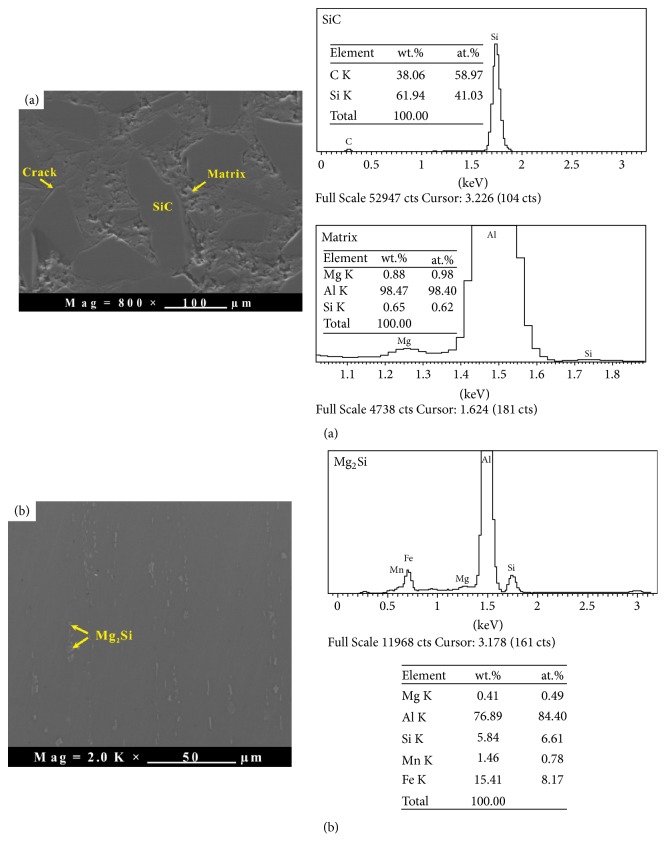
The morphologies of the SiC_p_/6061Al composite and the 6061 matrix. (a) Morphologies of the phases in the matrix of SiC_p_/6061Al and (b) intermetallic phase in the matrix of SiC_p_/6061Al composite.

The optical microstructures of the fabricated SiC_p_/6061Al composites with different volume fraction (namely, 60%, 65%, 70%, and 75%, respectively) of SiC particles are given in Figure 5. It is clear that the area fraction of the reinforcements in the microstructure is increased with the increase of SiC volume fraction. The reinforcements with different sizes are revealed in these microstructures, because the composites have been fabricated by using the SiC powder with the median diameters at 2 *μ*m, 8 *μ*m, and 128 *μ*m. Compared with Figure 5(d), Figures 5(a), 5(b), and 5(c) show the distribution of the reinforcements is relatively uniform. The agglomeration phenomenon on the small particles can be found in Figure 5(d). The morphologies of the SiC_p_/6061Al composite are characterized by SEM/EDS in Figure 6. It is clear that only a few cracks reveal at the boundaries of the large SiC particles (diameter > 100 *μ*m). But several voids still can be found in the matrix and some places of the boundaries. Obviously, the small particles (diameter < 10 *μ*m) are distributed in the matrix and the boundaries of the large SiC particles (see details in Figure 5). It can be deduced that the voids resulted from the agglomeration of the particles with low median diameter. The bonding of the agglomerated particles is weaker than the bonding property of SiC/matrix interface. It might lead to the peeling off of the particles during the polishing process of the SiC_p_/Al interface [10].

### 3.3. Sealing Interface of Bismuthate Glass and SiC_p_/6061Al Composites

**Figure 7:**
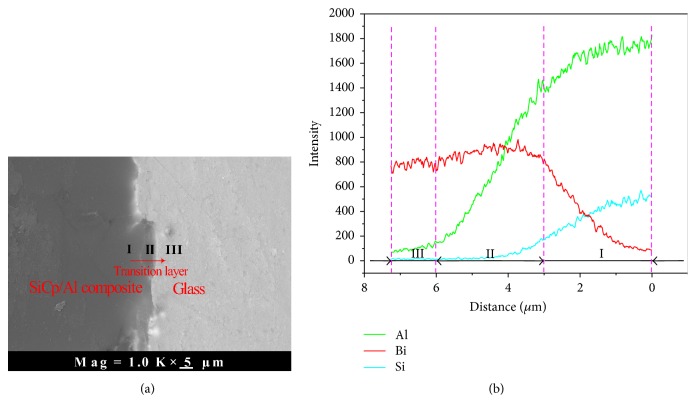
The interface micrograph and X-ray line scans obtained from the high volume fraction SiC_p_/Al and bismuthate glass.

Figure 7 shows the morphology of the SiC_p_/Al composite-bismuthate glass material at a magnification (1000x). The microstructure can be divided into three regions. As seen from the microstructure, Region I is the SiC_p_/Al composite which reveals the SiC reinforcements and the Al matrix. In this region, the concentration of Si element is high because of the uniformly distributed SiC particles. Region II is the transition layer, because the concentration of Bi element gradually changes with the various of the distance. Region III is the bismuthate glass. In this region, the concentration of Si is decreased, but the concentration of Bi element is stable. Moreover, it is found that the thickness of the transition layer (Region II) is about 9 *μ*m. The diffusion distance of these transition layer is obvious in the SiC_p_/Al composite-glass material interface, suggesting that strong chemical affinity existed between the glass and the composite [11].

### 3.4. CTE of SiC_p_/Al Composites and Bismuthate Glass

**Figure 8:**
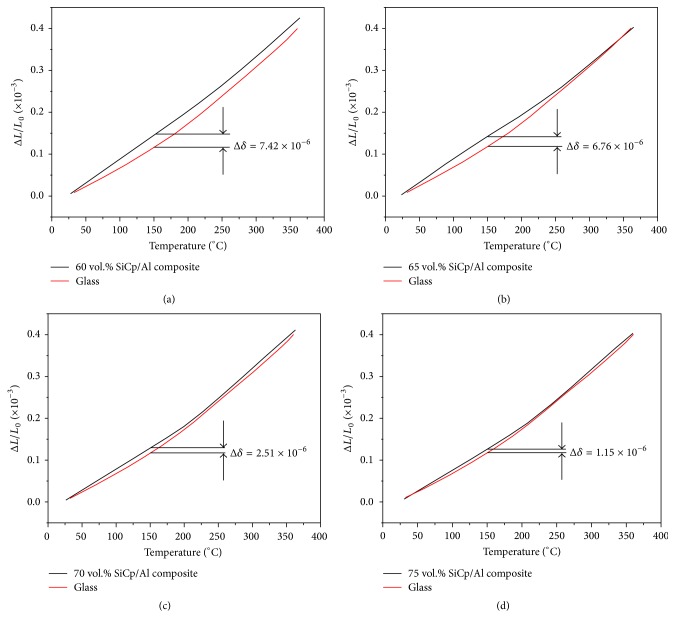
Thermal expansion coefficient comparison between high volume fraction SiC_p_/Al (60%, 65%, 70%, and 75%) and bismuthate glass.

**Figure 9:**
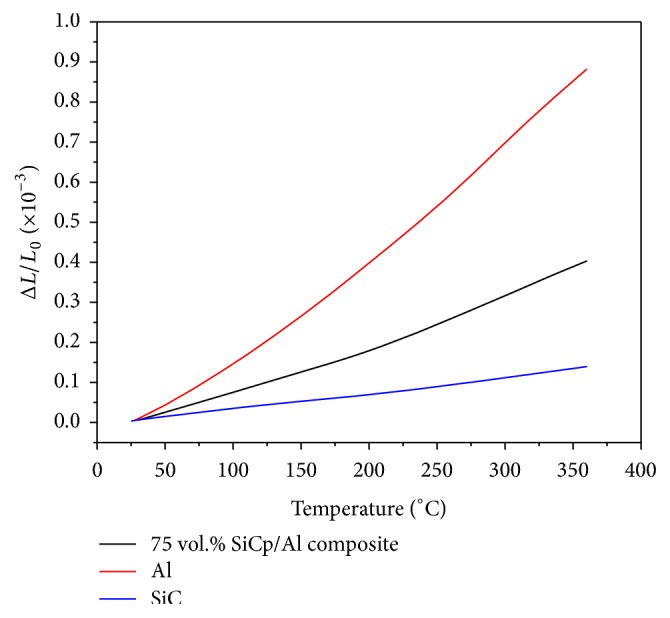
The relationship of the 75 vol.%  SiC_p_/Al composite with Al and SiC.

The CTE values (tested at 25°C) of bismuthate glass and the SiC_p_/6061Al composites with 60%, 65%, 70%, and 75% particles are listed in Table 3. It indicates that the CTE value of the in-house fabricated bismuthate glass is close to the composites with different fraction of SiC particles. However, the difference of CTE values between the bismuthate glass and the composites, represented by the symbol of Δ*δ* in Figure 8, is enlarged with the increase of CTE testing temperature. Compared with the composites with relatively lower SiC fraction (such as the 60%  SiC_p_/Al), the bigger fraction of the SiC leads to the lower Δ*δ* value. The CTE relationship of the 75 vol.%  SiC_p_/Al composite with Al and SiC is shown in Figure 9. The specific value is in Table 4.

### 3.5. Shear Strength of Bismuthate/Composite Interface

**Figure 10:**
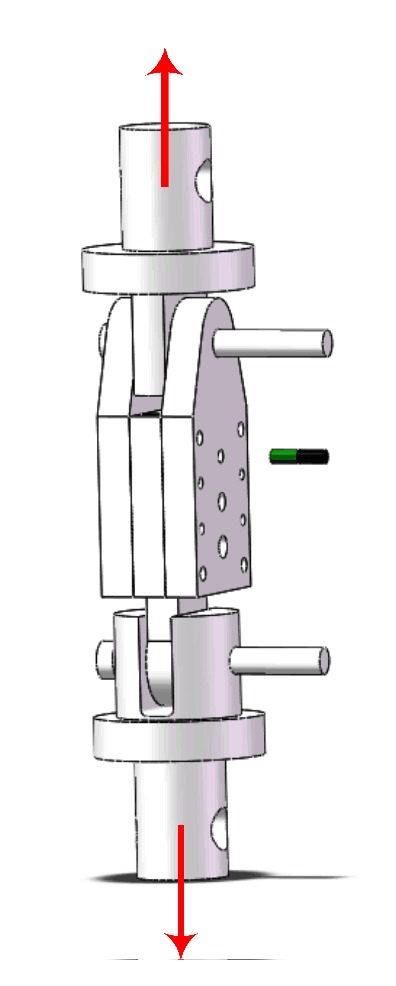
The schematic diagram of the test sample for shear strength.

**Figure 11:**
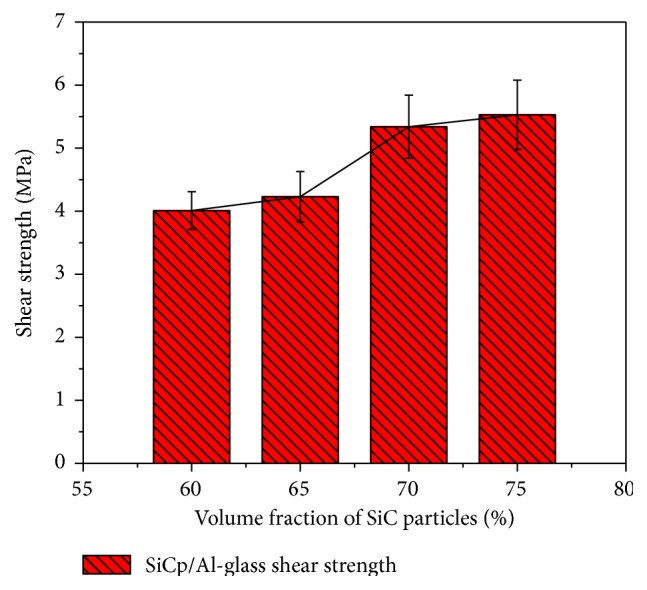
Shear strength morphology of the SiC_p_/Al composite-glass material.

The schematic diagram of the test sample for shear strength is given as shown in Figure 10. The shear strength of these sealed bismuthate glass-SiC_p_/Al composites samples (60%, 65%, 70%, and 75%) is concluded in Figure 11, which shows a remarkable increasing trend. The shear strength values of the 60% and 75% samples are 4.01 and 4.23 MPa, respectively. When the SiC fraction in the SiC_p_/Al composites increases to 70%, the shear strength suddenly increases to 5.34 MPa, which is 26% higher than that of the 65%  SiC_p_/Al sample. The shear strength of the 75%  SiC_p_/Al sample is 5.53 MPa, which is close to the value of the 70%  SiC_p_/Al sample.

**Figure 12:**
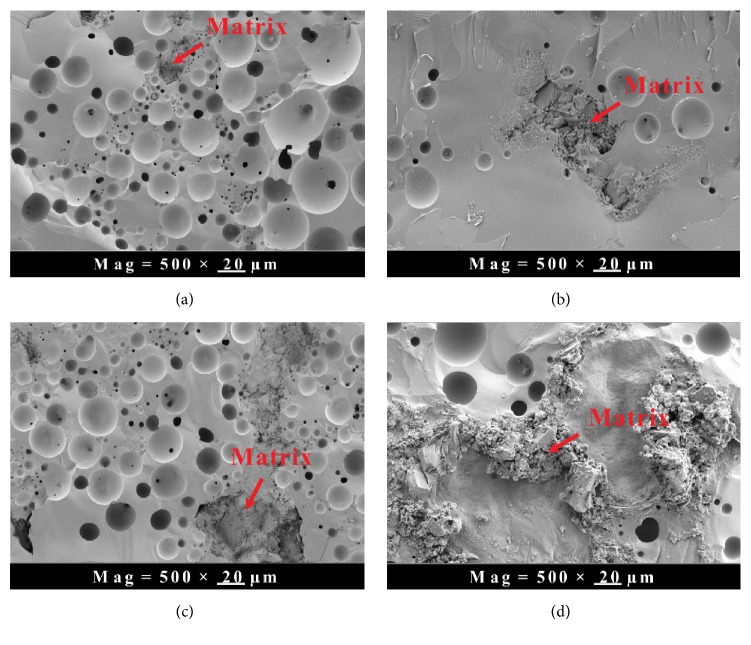
The fracture morphology of the composite-glass material.

The observation for the fracture surface is helpful to disclose the fracture mechanism of the shear strength samples.  Figure 12(a) shows the fracture surface of the sample fabricated by points out the composite matrix of the interface. Compared with area of the composite matrix in Figure 12(a), the corresponding area in Figure 12(b) is larger. Moreover, this area in Figure 12(c) is larger than that of Figure 12(b).  Figure 12(d) is the fracture surface of the sample fabricated by sealing the bismuthate glass and the 75%  SiC_p_/Al sample. In this fracture pattern, most of the area is the composite matrix. The content of SiC in the composite is vol.  60%, vol.% 65%, vol.% 70%, and vol.%  75% versus Figures 12(a), 12(b), 12(c), and 12(d), respectively.

## 4. Conclusions

(1) The bismuthate glass and the SiC_p_/Al composite were successfully sealed after preoxidizing the composite at 500°C with the holding time of 600 min in the air to obtain an Al_2_O_3_ layer.

(2) The line scanning result indicated the Bi element in bismuthate glass had been diffused into the SiC_p_/Al composite matrix.

(3) The higher the particle content was, the lower the CTE value was, which led to the high bonding strength of the sealing interface.

## Figures and Tables

**Table 1:** Chemical composition of 6061-Al.

Specification	Composition (wt%)							
	Cu	Mg	Fe	Si	Zn	Mn	Pb	Al
Aluminium alloy	0.26	1.10	0.23	0.96	0.24	0.17	0.09	96.95

**Table 2:** Chemical composition of bismuthate glass.

Bi_2_O_3_ (wt.%)	B_2_O_3_ (wt.%)	BaO (wt.%)	Li_2_O (wt.%)
72.70	15.00	10.00	2.30

**Table 3:** CTE values of the bismuthate glass and the SiC_p_/6061Al composites with different vol.% (tested at 25°C).

Materials	Bismuthate glass	60% SiC_p_/Al6061	65% SiC_p_/Al6061	70% SiC_p_/Al6061	75% SiC_p_/Al6061
CTE value	12.029 × *e*^−06^	13.6180 × *e*^−06^	12.9840 × *e*^−06^	12.1081 × *e*^−06^	12.053 × *e*^−06^

**Table 4:** CTE values of the SiC_p_/6061Al composite with Al and SiC (tested at 25°C).

Materials	SiC	Al	75% SiC_p_/Al6061
CTE value	4.722 × *e*^−06^	16.126 × *e*^−06^	12.053 × *e*^−06^
